# Spectrum and Clinical Reproductive Significance of Cytogenetic Abnormalities in Infertility and Recurrent Early Pregnancy Loss: A Five-Year Retrospective Study of 10,285 Cases

**DOI:** 10.7759/cureus.103325

**Published:** 2026-02-09

**Authors:** Suryaprakash Kunda, Mukkanteswararao Kasaragadda, Sambasivarao Patibandla, Shalini Singh, Sidrah Parvez, Tirupathi Rao Golla

**Affiliations:** 1 Cytogenetics Department, American Institute of Pathology and Laboratory Sciences (AMPATH) Labs, Hyderabad, IND; 2 Biotechnology Department, Eras Lucknow Medical College and Hospital, Lucknow, IND; 3 Conservation Genetics Lab, Council of Scientific and Industrial Research (CSIR) - Centre for Cellular and Molecular Biology, Hyderabad, IND

**Keywords:** infertility, karyotyping, recurrent early pregnancy loss, reproductive genetics, sex chromosome abnormalities

## Abstract

Introduction

Cytogenetic abnormalities are a major genetic cause of infertility and recurrent early pregnancy loss (REPL), conditions that affect a substantial proportion of couples of reproductive age. Both numerical and structural chromosomal abnormalities can impair gametogenesis, fertilization, implantation, or embryonic development. This study aimed to evaluate the frequency, spectrum, and clinical relevance of chromosomal abnormalities in individuals referred for infertility, single miscarriage, or recurrent pregnancy loss (RPL).

Materials and methods

This large retrospective cytogenetic study analyzed 10,285 individuals referred for infertility, single miscarriage, or recurrent pregnancy loss over a five-year period. Conventional G-banded karyotyping was performed in accordance with International System for Human Cytogenomic Nomenclature (ISCN) 2025 guidelines. Chromosomal abnormalities were classified as numerical or structural, with variants further categorized by specific chromosome involvement. Data on sex distribution and abnormality patterns were summarized to identify trends and clinically significant associations.

Results

Among the 10,285 individuals evaluated, cytogenetic abnormalities were identified in 980 cases, representing 9.5% of the study population. The cohort demonstrated a largely balanced sex distribution, with 46,XX karyotypes observed in 5,380 cases (52.3%) and 46,XY karyotypes in 4,857 cases (47.2%). Analysis of abnormal karyotypes revealed that chromosomal variants were the most frequent finding, accounting for 514 cases (52.4%), followed by variant inversions in 94 cases (9.6%). Structural chromosomal rearrangements were commonly observed and included reciprocal translocations in 120 cases (12.2%) and Robertsonian translocations in 33 cases (3.4%), involving multiple autosomes and sex chromosomes. Sex chromosome abnormalities comprised a notable subset, with Klinefelter syndrome identified in 53 cases (5.4%), Y-chromosome inversions in 36 cases (3.7%), mosaic karyotypes in 38 cases (3.9%), isochromosome X in 18 cases (1.8%), and additional X chromosomes in 12 cases (1.2%). Other abnormalities such as derivative chromosomes (24 cases, 2.4%), deletions (13 cases, 1.3%), autosomal inversions (14 cases, 1.4%), marker chromosomes (3 cases, 0.3%), and rare duplications or insertions (one case each, 0.1%) were observed less frequently, highlighting the broad spectrum of cytogenetic abnormalities associated with infertility and recurrent pregnancy loss.

Conclusion

This study demonstrates that chromosomal variants and structural rearrangements constitute the majority of cytogenetic abnormalities in individuals with infertility or recurrent pregnancy loss. Although clinically significant numerical and complex structural abnormalities were less frequent, they confer substantial reproductive risk through mechanisms affecting gametogenesis, chromosomal segregation, and embryonic viability.

## Introduction

Cytogenetics represents a core pillar of reproductive genetics, providing critical insight into the genomic abnormalities underlying infertility and recurrent early pregnancy loss (REPL). Early pregnancy loss affects approximately 15%-20% of clinically recognized pregnancies, and the occurrence of two or more losses defines REPL, affecting about 0.7%-1.9% of pregnancies worldwide and 1%-2% in the Indian population [1-3]. Although pregnancy loss is multifactorial, chromosomal abnormalities remain one of the most consistently implicated causes [4,5]. Cytogenetic defects account for approximately 2%-8% of recurrent pregnancy loss (RPL) in couples and up to 50%-70% of genetic abnormalities identified in products of conception [4,6]. Structural chromosomal rearrangements, particularly balanced reciprocal and Robertsonian translocations, represent the most frequent parental abnormalities [4,5,7]. Despite being phenotypically silent, these rearrangements disrupt meiotic segregation, resulting in unbalanced gametes and adverse reproductive outcomes [7,8]. Numerical chromosomal abnormalities, especially autosomal aneuploidies and sex chromosome defects, are the most common genetic causes of early miscarriage [6,9,10]. Sex chromosome aneuploidies and mosaicisms impair gametogenesis and gonadal function [9-11]. Furthermore, chromosomal polymorphisms and marker chromosomes, which were formerly considered to be benign, are increasingly recognized as context-dependent risk factors affecting reproductive outcomes [11-15]. Conventional G-banded karyotyping remains the diagnostic cornerstone for identifying balanced chromosomal rearrangements [7,16], while newer genomic technologies have expanded detection of cryptic imbalances [17]. Integrating classical cytogenetics with advanced genomic tools enhances diagnostic accuracy and reproductive counselling [16,17]. Therefore, the aim of this research was to assess the frequency, spectrum, and clinical significance of chromosomal abnormalities among individuals diagnosed with infertility, single miscarriage, or recurrent pregnancy loss.

## Materials and methods

This retrospective descriptive cytogenetic study was conducted over a five-year period at AMPATH Labs, Hyderabad, Telangana, India, a high-volume tertiary referral diagnostic laboratory. The study involved a systematic analysis of peripheral blood karyotyping results obtained as part of routine clinical care from individuals referred for evaluation of infertility, single miscarriage, or recurrent pregnancy loss (RPL). Cytogenetic data collected between January 2020 and October 2025 were included, representing a continuous period of referrals.

A total of 10,285 individuals were included in the analysis. The study population comprised both male and female partners of couples referred for evaluation of primary or secondary infertility, single pregnancy loss, or recurrent pregnancy loss. Referrals were made by reproductive medicine specialists, obstetricians, gynecologists, and infertility clinicians based on clinical suspicion of underlying chromosomal abnormalities. Only cases with complete cytogenetic records and analyzable metaphase preparations were included.

This study involved retrospective analysis of existing laboratory data generated during routine diagnostic evaluation. The study protocol was reviewed and approved by the Institutional Ethics Committee of AMPATH Labs, India (approval number: IRC/AMPATH/02/2026). All patient identifiers were removed prior to data extraction and analysis, and strict confidentiality was maintained throughout the study. As no direct patient contact, intervention, or additional sample collection was undertaken for research purposes, the requirement for informed consent was waived by the ethics committee.

Peripheral venous blood samples (approximately 2 mL) were collected aseptically in sodium heparin vacutainer tubes. Samples were transported at room temperature and processed within the recommended time frame to ensure optimal lymphocyte viability. Chromosome analysis was performed using the standard peripheral blood lymphocyte culture technique with minor laboratory-specific modifications based on the classical protocol described by Moorhead et al. (1960) [18]. Briefly, 0.5 mL of heparinized whole blood was added to RPMI-1640 culture medium supplemented with fetal bovine serum, antibiotics, and phytohemagglutinin to stimulate T-lymphocyte proliferation. Duplicate cultures were established for each sample and maintained until reporting. Cultures were incubated at 37°C in a humidified atmosphere for 72 hours.

Mitotic arrest was achieved by adding colchicine (1%) approximately 60-90 minutes prior to harvest. Cells were subsequently subjected to hypotonic treatment using 0.075 M potassium chloride to induce nuclear swelling and facilitate optimal chromosome spreading. Hypotonic treatment conditions were carefully controlled with respect to timing and temperature to minimize chromosome loss or overlap. Following hypotonic treatment, cells were fixed using freshly prepared methanol-acetic acid (3:1) fixative, with multiple fixation washes performed to ensure complete removal of cytoplasmic debris, in accordance with the method described by Hungerford (1965) [19]. Fixed cell suspensions were dropped onto clean, chilled glass slides to obtain well-spread metaphases. Slides were air-dried and appropriately aged prior to banding.

Chromosome banding was performed using the Giemsa-Trypsin-Giemsa (GTG) banding technique following the method described by Seabright (1971) [20]. Slides were briefly treated with trypsin to partially digest chromosomal proteins and subsequently stained with Giemsa, producing reproducible light and dark banding patterns along the chromosomes. Banding resolution was optimized to allow accurate identification of both numerical and structural chromosomal abnormalities.

Metaphase spreads from both duplicate cultures were analyzed using a bright-field microscope equipped with an IKAROS imaging (MetaSystems, Altlussheim, Germany) and a karyotyping system (Carl Zeiss, Jena, Germany). For each sample, 20-25 metaphases were routinely examined. In cases with suspected sex chromosome abnormalities, at least 30 metaphases were analyzed, while a minimum of 50 metaphases were evaluated in cases where mosaicism was suspected. Independent slide preparation and karyotype analysis were performed by two trained cytogenetic technologists to minimize observer bias and technical variability.

Karyotypes were interpreted and reported in accordance with the International System for Human Cytogenomic Nomenclature (ISCN) guidelines applicable at the time of analysis, with nomenclature harmonized to the most recent ISCN 2025 recommendations. Chromosomal abnormalities were classified as numerical abnormalities, structural abnormalities, chromosomal variants or heteromorphisms, mosaic karyotypes, and marker chromosomes.

Strict internal quality control measures were followed throughout the study period, including duplicate culture setup and slide evaluation, periodic inter-observer concordance checks, use of standardized reagents and calibrated equipment, and re-analysis of ambiguous or borderline findings. Demographic details, referral indications, sex distribution, and cytogenetic findings were extracted from laboratory records and entered into a structured database. All data were anonymized prior to analysis.

Given the retrospective and descriptive nature of the study, data were summarized using frequencies and percentages. Sex distribution and patterns of chromosomal abnormalities were analyzed descriptively. No inferential statistical tests were applied. Statistical analysis was performed using standard spreadsheet and statistical software using SPSS Statistics version 28.0 software (IBM Inc., Armonk, NY).

## Results

A total of 10,285 individuals were included in the analysis. Of these, 4,857 (47.22%) had a 46,XY karyotype and 5,380 (52.31%) had a 46,XX karyotype (Table 1), demonstrating a largely balanced sex distribution with a slight predominance of 46,XX karyotypes. This distribution is comparable to those reported in large infertility and recurrent pregnancy loss cohorts [4,5,12,21].

**Table 1:** Distribution of 46,XX and 46,XY karyotypes in the study cohort (N=10,285)

Serial number	Gender	Number (%)
1	46,XY	4,857 (47.2%)
2	46,XX	5,380 (52.3%)
Total		10,285 (100%)

Overall spectrum of cytogenetic abnormalities

Among the total cohort, cytogenetic abnormalities were identified in 980 cases (9.5%). Analysis of these abnormal karyotypes revealed a broad spectrum of numerical and structural chromosomal abnormalities. Chromosomal variants were the most prevalent category, accounting for 514 cases (52.4% of abnormal karyotypes), followed by variant inversions in 94 cases (9.6%), consistent with previously reported cytogenetic patterns in infertility and RPL populations [12-14,22].

Structural chromosomal rearrangements constituted a substantial proportion of abnormalities and included reciprocal translocations in 120 cases (12.2%) and Robertsonian translocations in 33 cases (3.4%). In addition, derivative chromosomes were identified in 24 cases (2.4%), autosomal inversions (excluding the Y chromosome) in 14 cases (1.4%), and Y-chromosome inversions in 36 cases (3.7%), in agreement with earlier cytogenetic studies [4,5,7,8,23].

Chromosomal variants

Among the 514 chromosomal variant cases, Y-chromosome heterochromatic variants were the most frequent, with Yqh+ observed in 155 cases (30.2%) and Yqh- in 34 cases (6.6%). Variants involving chromosome 9 were identified in 131 cases (25.5%), followed by chromosome 15 in 52 cases (10.1%). Variants of chromosomes 21 and 22 were observed in 29 cases (5.6%) and 31 cases (6%), respectively, while chromosome 1 variants accounted for 25 cases (4.9%) (Table 2).

**Table 2:** Distribution of chromosomal variants

Serial number	Chromosomes	Number of cases	Total	%
1	1	25	514	4.86
2	9	131	514	25.49
3	13	10	514	1.95
4	14	23	514	4.47
5	15	52	514	10.12
6	16	23	514	4.47
7	18	1	514	0.19
8	21	29	514	5.64
9	22	31	514	6.03
10	Yqh-	34	514	6.61
11	Yqh+	155	514	30.16

This distribution parallels findings from studies reporting increased frequencies of heterochromatic polymorphisms in reproductive failure cohorts [12-15].

Robertsonian translocations

A total of 33 Robertsonian translocation carriers (3.4% of abnormal cases) were identified. The most frequent rearrangement was rob(13;14), observed in 24 cases (72.7%), followed by rob(14;21) in five cases (15.2%) and rob(21;21) in two cases (6.1%). Rare rearrangements such as rob(14;14) and rob(21;22) were each identified in one case (3%), consistent with global and Indian cytogenetic literature (Table 3) [4,7,8,23].

**Table 3:** Distribution of cytogenetic abnormalities among abnormal karyotypes identified in individuals evaluated for infertility and recurrent pregnancy loss

Serial number	Abnormal	Sum of the number of cases	%
1	Additional	6	0.61%
2	Deletion	13	1.33%
3	Derivative	24	2.45%
4	Duplication	1	0.10%
5	Insertion	1	0.10%
6	Inversion	14	1.43%
7	Inversion Y	36	3.67%
8	Iso chromosome X	18	1.84%
9	Klinefelter	53	5.41%
10	Marker	3	0.31%
11	Mosaic	38	3.88%
12	Robertsonian	33	3.37%
13	Translocation	120	12.24%
14	Variant	514	52.45%
15	Variant inversion	94	9.59%
16	XXX	5	0.51%
17	XXXX	1	0.10%
18	XXXXX	1	0.10%
19	XXY	3	0.31%
20	XYY	2	0.20%
		980	

Normal variants of chromosome 9 and the Y chromosome

A total of 130 cases involved variants of chromosome 9 and the Y chromosome. Pericentric inversion of chromosome 9 [inv(9)] was observed in 94 cases (72.3%), while Y chromosome inversion variants were detected in 36 cases (27.7%). Similar predominance of inv(9) and Y chromosome heteromorphisms has been reported in infertility and recurrent miscarriage cohorts [13-15].

Sex chromosome abnormalities

Sex chromosome abnormalities were identified in 83 cases (8.5% of abnormal karyotypes). Klinefelter syndrome (47,XXY) was the most frequent abnormality, observed in 53 cases (63.9%). Isochromosome X was detected in 18 cases (21.7%), while other abnormalities included 47,XXX in five cases (6%), XYY in two cases (2.4%), XXY variants in four cases (4.8%), and rare polysomies (48,XXXX and 49,XXXXX) in one case each (1.2%). These findings align with previous reports on the contribution of sex chromosome abnormalities to infertility and recurrent pregnancy loss [9-11].

Inversions

A total of 112 inversion cases were identified, involving multiple autosomes. The most frequent inversion involved chromosome 22 in 22 cases (19.6%), followed by chromosome 8 in 24 cases (21.4%), chromosome 18 in 18 cases (16.1%), and chromosome 17 in 17 cases (15.2%). Inversions involving chromosomes 6, 4, and 7 were also observed, reflecting the heterogeneity of inversion breakpoints reported in reproductive failure cohorts [8,24].

Derivative chromosomes and other structural abnormalities

Derivative chromosomes were identified in 24 cases (2.4%), involving multiple autosomes and the X chromosome. Other structural abnormalities included chromosomal additions, insertions, duplications, and deletions, which generally occurred as isolated findings. Deletions were identified in 13 cases (1.3%), while duplications and insertions were each observed in one case (0.1%). These abnormalities have similarly been associated with adverse reproductive outcomes in previous studies [6,16].

Translocations

Reciprocal translocations involved almost all autosomes and the sex chromosomes and included two-way, three-way, and four-way translocations. Frequent involvement of chromosomes 1, 4, 6, 11, 13, and acrocentric chromosomes mirrors patterns reported in earlier cytogenetic analyses of infertility and RPL (Table 4) [4,5,8].

**Table 4:** Distribution of chromosomes involved in reciprocal translocations identified among abnormal karyotypes in individuals evaluated for infertility and recurrent pregnancy loss

Serial number	Chromosome	Number of cases
1	1	15
2	2	13
3	3	7
4	4	18
5	5	5
6	6	10
7	7	5
8	8	5
9	9	4
10	10	6
11	11	7
12	12	4
13	13	3
14	14	3
15	15	2
16	16	2
17	18	3
18	20	1
19	X	5
20	Y	2

Mosaic karyotypes were identified in 38 cases (3.9%), involving both autosomes and sex chromosomes, with common patterns including sex chromosome mosaicism and isochromosome X. Marker chromosomes were identified in three cases (0.3%), occurring as isolated or mosaic findings. These observations further support chromosomal instability as an important mechanism contributing to reproductive failure [11].

## Discussion

The interpretation and clinical relevance of the cytogenetic findings observed in this study are supported by multiple international and Indian cohort studies, clinical practice guidelines, and authoritative reviews. The contribution of chromosomal abnormalities to infertility and recurrent pregnancy loss has been well documented in large cytogenetic studies and systematic evaluations [4-6,12,21].

The clinical significance of balanced structural rearrangements, including reciprocal and Robertsonian translocations, and their association with abnormal meiotic segregation and adverse reproductive outcomes have been consistently demonstrated in earlier studies and reviews [4,5,7,8,23]. Current international guidelines from professional societies further emphasize the importance of parental karyotyping in couples with recurrent pregnancy loss [1,2].

Chromosomal variants and heteromorphisms, particularly those involving chromosome 9 and Y chromosome heterochromatin, have been increasingly reported at higher frequencies in infertility and recurrent pregnancy loss cohorts compared with the general population, suggesting a potential modifying or predisposing role in reproductive failure [12-15].

Sex chromosome abnormalities, including Klinefelter syndrome, isochromosome X, and other sex chromosome aneuploidies, are well-established causes of impaired gametogenesis, gonadal dysfunction, and pregnancy loss [9,10]. Mosaic karyotypes and marker chromosomes further highlight chromosomal instability as an important pathogenic mechanism in reproductive failure [11].

In addition, complex chromosomal rearrangements and inversions have been shown to markedly increase reproductive risk due to abnormal meiotic behavior and high rates of unbalanced gamete formation [8,24]. Authoritative cytogenetic texts and counseling guidelines support careful interpretation of such findings and stress the importance of individualized genetic counseling [16]. Emerging genomic technologies, including optical genome mapping, further complement conventional cytogenetics by improving the detection of cryptic rearrangements in recurrent pregnancy loss [17].

This study has certain limitations that should be acknowledged. First, the retrospective design limits control over clinical and demographic variables and precludes causal inference between specific cytogenetic abnormalities and reproductive outcomes. Detailed clinical information, such as gestational age at pregnancy loss, number of losses, assisted reproductive treatment history, and longitudinal reproductive outcomes, was not uniformly available for all cases.

Second, the study relied primarily on conventional G-banded karyotyping, which is the diagnostic standard for detecting numerical abnormalities and balanced structural rearrangements but has limited resolution for identifying submicroscopic chromosomal imbalances or gene-level alterations. As a result, cryptic copy number variants and single-gene defects contributing to infertility or recurrent pregnancy loss may not have been detected. Advanced genomic techniques such as chromosomal microarray analysis or optical genome mapping were not routinely applied.

Third, the cohort consisted of individuals referred to a tertiary diagnostic center, which may introduce referral bias and limit the generalizability of the findings to the broader population. Additionally, the absence of a fertile control group restricts direct comparison of chromosomal variant frequencies with those in the general population.

Finally, although karyotypes were interpreted using ISCN guidelines applicable at the time of testing and harmonized to current nomenclature for reporting, minor differences across ISCN updates may have influenced the classification of certain findings. Nevertheless, these limitations are unlikely to affect the overall conclusions of the study.

Despite these limitations, the large sample size, standardized cytogenetic methodology, and comprehensive classification of abnormalities provide robust insights into the spectrum and reproductive significance of cytogenetic alterations in infertility and recurrent pregnancy loss.

## Conclusions

This large retrospective study demonstrates that cytogenetic abnormalities remain a major genetic contributor to infertility and recurrent early pregnancy loss. The high frequency of chromosomal variants, particularly inv(9) and Yqh+, suggests their possible role as predisposing or modifying factors in adverse reproductive outcomes. Additionally, the identification of structural rearrangements highlights the increased risk of unbalanced gamete formation and miscarriage. Overall, these findings support the continued use of comprehensive cytogenetic analysis as a first-line tool for diagnosis, genetic counseling, and informed reproductive decision-making in affected couples.

## References

[1] Fauser BC, Adamson GD, Boivin J (2024). Declining global fertility rates and the implications for family planning and family building: an IFFS consensus document based on a narrative review of the literature. Hum Reprod Update.

[2] (2020). Definitions of infertility and recurrent pregnancy loss: a committee opinion. Fertil Steril.

[3] Turesheva A, Aimagambetova G, Ukybassova T (2023). Recurrent pregnancy loss etiology, risk factors, diagnosis, and management. Fresh look into a full box. J Clin Med.

[4] Franssen MT, Korevaar JC, van der Veen F, Leschot NJ, Bossuyt PM, Goddijn M (2006). Reproductive outcome after chromosome analysis in couples with two or more miscarriages: index [corrected]-control study. BMJ.

[5] Aynaci S, Kocagil S, Tosumoglu E (2025). Chromosomal abnormalities in couples with recurrent pregnancy loss: a 16-year cross-sectional study of 4030 cases from Turkey. Ann Saudi Med.

[6] Pande S, Babu S, Gawde H, Minde N (2024). Repeated pregnancy losses with multiple aneuploidies and major genomic imbalance: a case report. Asian Pac J Reprod.

[7] Madan K (2012). Balanced complex chromosome rearrangements: reproductive aspects. A review. Am J Med Genet A.

[8] Pellestor F, Anahory T, Lefort G, Puechberty J, Liehr T, Hédon B, Sarda P (2011). Complex chromosomal rearrangements: origin and meiotic behavior. Hum Reprod Update.

[9] Lanfranco F, Kamischke A, Zitzmann M, Nieschlag E (2004). Klinefelter's syndrome. Lancet.

[10] Gravholt CH, Andersen NH, Christin-Maitre S (2024). Clinical practice guidelines for the care of girls and women with Turner syndrome. Eur J Endocrinol.

[11] Spinella F, Fiorentino F, Biricik A (2018). Extent of chromosomal mosaicism influences the clinical outcome of in vitro fertilization treatments. Fertil Steril.

[12] Popescu-Hobeanu G, Serban Sosoi S, Cucu M (2024). The value of parental karyotyping in recurrent pregnancy loss lies in individual risk assessments. Medicina (Kaunas).

[13] Merrion K, Maisenbacher M (2019). Pericentric inversion (Inv) 9 variant-reproductive risk factor or benign finding?. J Assist Reprod Genet.

[14] Kosyakova N, Grigorian A, Liehr T (2013). Heteromorphic variants of chromosome 9. Mol Cytogenet.

[15] Krausz C, Abrardo C (2025). The Y chromosome: male reproduction and beyond. Fertil Steril.

[16] Sharma A, Minhas S, Dhillo WS, Jayasena CN (2021). Male infertility due to testicular disorders. J Clin Endocrinol Metab.

[17] Del Águila MD, Bernal M, Vílchez JR (2025). Optical genome mapping enhances cytogenetic analysis in recurrent miscarriage: confirmation of a suspected (1;10) chromosomal translocation. Mol Cytogenet.

[18] Moorhead PS, Nowell PC, Mellman WJ, Battips DM, Hungerford DA (1960). Chromosome preparations of leukocytes cultured from human peripheral blood. Exp Cell Res.

[19] Hungerford DA (1965). Leukocytes cultured from small inocula of whole blood and the preparation of metaphase chromosomes by treatment with hypotonic KCl. Stain Technol.

[20] Seabright M (1971). A rapid banding technique for human chromosomes. Lancet.

[21] Sinha MB, Thakur P, Verma R (2025). Chromosomal abnormalities in recurrent pregnancy loss at a tertiary care center. Cureus.

[22] Bhattacharjee K, Pal S, Banerjee E, Mukherjee S (2024). Recurrent pregnancy loss associated cytogenetic and genetic anomalies - study from eastern India. Int J Exp Res Rev.

[23] Lu W, Zhou J, Rao H, Yuan H, Huang S, Liu Y, Yang B (2024). A retrospective analysis of Robertsonian translocations from a single center in China. Reprod Sci.

[24] Antonelli A, Gandini L, Petrinelli P (2000). Chromosomal alterations and male infertility. J Endocrinol Invest.

